# Vehicle-as-a-Sensor Approach for Urban Track Anomaly Detection

**DOI:** 10.3390/s25216679

**Published:** 2025-11-01

**Authors:** Vlado Sruk, Siniša Fajt, Miljenko Krhen, Vladimir Olujić

**Affiliations:** 1 University of Zagreb Faculty of Electrical Engineering and Computing, Unska 3, 10000 Zagreb, Croatia; sinisa.fajt@fer.unizg.hr (S.F.); vladimir.olujic2@fer.unizg.hr (V.O.); 2 University of Zagreb Faculty of Textile Technology, Prilaz baruna Filipovića 28a, 10000 Zagreb, Croatia; miljenko.krhen@ttf.unizg.hr

**Keywords:** vibration-based monitoring, anomaly detection, urban tram infrastructure, predictive maintenance, vehicle-as-a-sensor

## Abstract

This paper presents a Vibration-based Track Anomaly Detection (VTAD) system designed for real-time monitoring of urban tram infrastructure. The novelty of VTAD is that it converts existing public transport vehicles into distributed mobile sensor platforms, eliminating the need for specialized diagnostic trains. The system integrates low-cost micro-electro-mechanical system (MEMS) accelerometers, Global Positioning System (GPS) modules, and Espressif 32-bit microcontrollers (ESP32) with wireless data transmission via Message Queuing Telemetry Transport (MQTT), enabling scalable and continuous condition monitoring. A stringent ±6σ statistical threshold was applied to vertical vibration signals, minimizing false alarms while preserving sensitivity to critical faults. Field tests conducted on multiple tram routes in Zagreb, Croatia, confirmed that the VTAD system can reliably detect and locate anomalies with meter-level accuracy, validated by repeated measurements. These results show that VTAD provides a cost-effective, scalable, and operationally validated predictive maintenance solution that supports integration into intelligent transportation systems and smart city infrastructure.

## 1. Introduction

Urban transportation systems, including both road and tram infrastructure, are fundamental to modern society providing mobility, supporting economic activity, and contributing to sustainable urban development [1]. These systems facilitate daily commuting for a large proportion of the population and are increasingly recognized as beneficial for the environment [2]. However, the effectiveness of public transportation is inextricably linked to its safety, reliability and passenger comfort, all of which are heavily dependent on the condition of the underlying infrastructure, particularly the tracks [3]. Consequently, the continuous monitoring of operational anomalies and mechanical faults is of paramount importance [4]. Faults occurring in any segment of these systems must be detected and rectified immediately to prevent service interruptions, potential accidents and the long-term degradation of the infrastructure due to undetected faults [5]. This requires the development of advanced monitoring systems capable of assessing and logging deviations from expected operating parameters in real time [6].

Maintenance of tram infrastructure has traditionally relied on manual inspections by technical staff or the use of specialized diagnostic vehicles. Manual inspections require the use of specialized instruments to monitor the condition of the track, while diagnostic vehicles are highly accurate and effective, but expensive and complex to operate [7]. This highlights the need for a more cost-effective and compact solution that still provides sufficient accuracy and diagnostic confidence [7,8]. The demand for such innovative approaches is exacerbated by increasing urbanization and traffic congestion in cities worldwide, which necessitate efficient and sustainable transportation systems [9]. The high implementation costs of advanced intelligent transportation system, including extensive sensor networks and communication systems, remain a major challenge and are driving the search for more economical deployment strategies [10]. The need for continuous monitoring of smart tram infrastructure has also been highlighted in recent critical reviews [11], emphasizing the shift towards more persistent and automated assessment methods [12,13].

Recent advances in low-cost sensing technologies have significantly improved the feasibility of track structure health monitoring. In particular, micro-electro-mechanical system (MEMS)-based accelerometers and inertial sensors have demonstrated strong potential for practical applications in both railway and road transport systems. For example, MEMS sensors have been applied for evaluating cargo dynamics and securing conditions in road vehicles, showing robustness under real operating environments [14]. In the railway sector, a number of studies have proposed and validated onboard monitoring systems for track condition assessment. An in-service train monitoring system was developed and implemented [15], while onboard sensor-based measurements were compared with conventional track recording data, showing good agreement in geometry monitoring [16]. The use of MEMS accelerometers for monitoring specific track components has been widely examined. MEMS devices installed on railway bearers at switches and crossings have been shown to effectively capture local displacements [17]. Experimental tests on various low-cost inertial devices have demonstrated their suitability for both ride comfort assessment and track condition monitoring, supporting their applicability for large-scale use [18]. A method for estimating track irregularities by combining multiple MEMS-IMUs with geometric constraints has been developed, achieving enhanced accuracy [19]. Additionally, a data-driven technique utilizing car body vibration for irregularity estimation has been implemented, further confirming the potential of low-cost sensors in practical monitoring scenarios [20].

Smartphones and other mobile devices have also been investigated as promising platforms for cost-effective track monitoring. Smartphone-based sensing for on-board railway monitoring has been applied, demonstrating the capability to assess structural performance and geometrical degradation [21]. This approach has been further extended to the analysis of ride comfort and transition zones using smartphones and tablets [22], while a methodology and field results for tram transport using mobile devices for acceleration measurement have also been presented [23]. These developments highlight the increasing role of low-cost MEMS sensors and consumer-grade devices in vibration-based monitoring of track structures.

In response to these challenges, this paper presents a conceptual and validated vehicle-mounted system, referred to as the Vibration-Based Track Anomaly Detection (VTAD) system, designed for the rapid detection of potentially hazardous anomalies in tramway tracks. The VTAD system employs software-based signal analysis to identify abnormal vibration patterns and accurately localize suspected faults. Its core principle is to transform existing public transportation vehicles into mobile inspection units, thereby creating a distributed sensor network that leverages the mobility of trams. This approach provides tramway operators with a cost-effective tool for continuous condition monitoring and proactive maintenance. With further development, VTAD could be scaled to cover entire tramway networks, forming the foundation for a large-scale, real-time infrastructure health monitoring system.

Building on the existing literature [24,25], this paper makes several specific contributions to the field of urban infrastructure monitoring. First, it presents a novel, low-cost, and modular vibration-based system that leverages existing public transit vehicles as mobile, distributed, real-time sensor platforms—as enabler of shift from traditional, costly diagnostic approaches that rely on dedicated inspection equipment. Second, a robust statistical anomaly detection method with a stringent ±6σ threshold is introduced and validated, which is used to minimize the number of false alarms in critical infrastructure assessment. Third, this study presents empirical validation through extensive field testing on various types of urban tram routes in Zagreb, Croatia, demonstrating the system’s ability to reliably locate track anomalies with high spatial accuracy through repeated measurements. This approach is designed to provide a highly scalable and operationally integrated solution for possible continuous monitoring of infrastructure conditions, thereby supporting proactive maintenance ab extend smart city ecosystems.

## 2. VTAD System Architecture and Hardware Components

The proposed VTAD system is modular and scalable and can be seamlessly integrated into a variety of microcontroller-based platforms. This architecture was deliberately chosen to enable cost-effective real-time monitoring of transportation infrastructure. The inherent modularity and scalability of the system directly address the economic and technical complexities often associated with large-scale infrastructure monitoring solutions, making it a commercially viable option for widespread deployment. With simpler integration and lower unit costs, this design supports the transformation of urban vehicle fleets into large-scale distributed sensor networks.

The VTAD system was prototyped on the Arduino platform and later built on the Espressif 32-bit microcontroller (ESP32) platform, utilizing its built-in Wi-Fi capability. This architectural change from the traditional Arduino Uno improves the real-time processing performance of the system and enables wireless data transmission via the Message Queuing Telemetry Transport protocol, which is essential for scalable deployment in urban environments.

The hardware configuration consists of three main components: an ESP32 development board, a 10 degrees-of-freedom inertial measurement unit (IMU) and a Global Positioning System (GPS) module. These components are integrated to form a distributed measurement node that collects vibration and position data and transmits it wirelessly to a central monitoring platform. This flexible architecture enables the strategic deployment of sensors on different types of vehicles, including tram and road vehicles, so that they can act as mobile control units on their regular operational routes [7].

**Figure 1:**
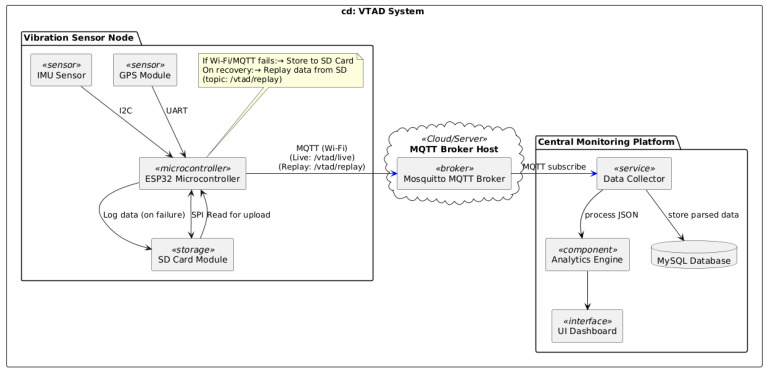
Vibration-Based Track Anomaly Detection (VTAD) system Unified Modeling Language (UML) Component Diagram.

Unified Modeling Language (UML) Component Diagram presented in Figure 1 outlines the system architecture. The system comprises three core logical components: the Vibration Sensor Node, the Mosquitto MQTT Broker, and the Central Monitoring Platform. Vibration Sensor Node: Deployed on vehicles, this node integrates an ESP32 with an IMU Sensor for vibration data, a GPS Module for location, and an SD Card Module for local data storage. The ESP32 publishes live data to the MQTT Broker via Wi-Fi. Crucially, if Wi-Fi/MQTT connectivity fails, data is logged to the SD Card and replayed for upload upon network recovery, ensuring data resilience. Mosquitto Message Queuing Telemetry Transport (MQTT) Broker facilitates real-time, publish-subscribe communication, receiving data from sensor nodes and distributing it to the Central Monitoring Platform. Central Monitoring Platform processes and stores the collected data. It includes a Data Collector Service that subscribes to the MQTT Broker, receiving both live and replayed data, and stores this raw information in a MySQL Database.

This architecture emphasizes robust data collection, resilient transmission, and centralized storage for effective anomaly detection.

### 2.1. ESP32 Microcontroller Unit

The ESP32 serves as a central processing and communication unit [26]. It has a 32-bit dual-core central processing unit operating at 240 MHz, 520 KB SRAM and up to 4 MB flash memory. The board supports several UART, I^2^C and SPI interfaces and thus enables flexible sensor connection. One core of the ESP32 is responsible for real-time data acquisition from the IMU and GPS modules, while the other handles MQTT-based data transmission via Wi-Fi.

The ESP32 is programmed using the Arduino IDE with the PubSubClient library for MQTT and Wire.h for I^2^C communication. Its integrated Wi-Fi stack enables seamless connection to a local network or hotspot so that sensor data can be published to a cloud-based MQTT broker Mosquitto at regular intervals. To increase the system’s resilience to unreliable networks, a SparkFun Thing Plus—ESP32-S3 development board [27] is utilized, specifically chosen for its integrated SD card slot. This allows for conditional SD logging: if the MQTT client is not connected to the broker, or if a client.publish() call fails due to a temporary network disruption, the sensor data is immediately written to the SD card. This mechanism ensures that the data is always preserved by either transferring it to the cloud via MQTT or logging it locally, thereby making the system robust against disruptions.

### 2.2. Inertial Measurement Unit

The sensor used is a 10-DOF IMU board, which integrates a 9-DOF MPU9255 (3-axis accelerometer, 3-axis gyroscope, 3-axis magnetometer) and a BMP180 pressure sensor for altitude. For this study, our analysis focused exclusively on the data from the 3-axis accelerometer to capture the vehicle’s vibration signature along the X, Y and Z axes at a sampling rate of 60 Hz. The IMU communicates with the ESP32 via the I^2^C bus using the SDA and SCL lines. The sensor used is a 10-DOF IMU board, which integrates a 9-DOF MPU9255 (3-axis accelerometer, 3-axis gyroscope, 3-axis magnetometer) [28] and a BMP180 pressure sensor for altitude. For this study, our analysis focused exclusively on the data from the 3-axis accelerometer to capture the vehicle’s vibration signature. The MPU9255 component captures this signature along the X, Y, and Z axes at a sampling rate of 60 Hz and communicates with the ESP32 via the I^2^C bus.

The MPU9255 was selected for this application due to its combination of high resolution, proven reliability, and low cost. The accelerometer features a 16-bit analog-to-digital converter (ADC), which provides the high-resolution data necessary for this study. For our measurements, we configured the sensor to a full-scale range of ±4 g, resulting in a sensitivity of 8192 LSB/g. This level of precision is crucial for capturing the subtle variations in vibration signatures needed for early-stage anomaly detection, thus qualifying it as a high-resolution sensor in the context of MEMS devices suitable for this application.

**Figure 2:**
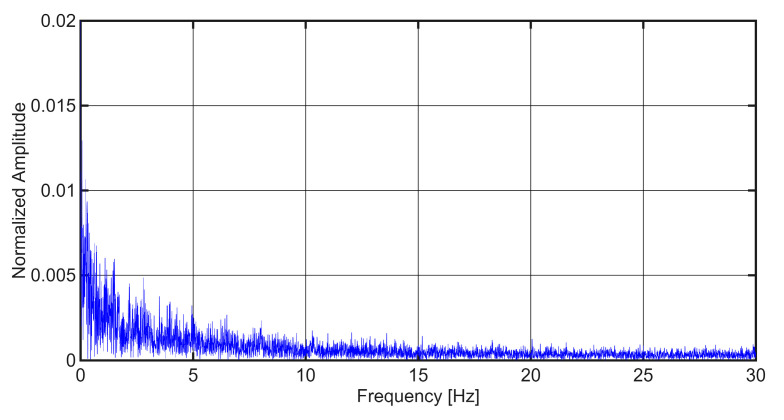
Fast Fourier Transform (FFT) spectrum of the recorded vertical vibration signal from the VTAD.

A frequency-domain analysis was conducted to confirm the suitability of the 60 Hz sampling frequency. The power spectrum of the vertical vibration signal, derived using a Fast Fourier Transform (FFT) for Case Study 3, is presented in Figure 2. The analysis shows a clear concentration of signal energy at frequencies below 10 Hz, with negligible content beyond 20 Hz. Given the corresponding Nyquist frequency of 30 Hz, the selected sampling rate is demonstrably adequate for preventing aliasing and ensuring the complete capture of vibration phenomena associated with track irregularities.

The choice of a high-resolution IMU with digital output simplifies synchronization and reduces analog noise. The data is pre-processed onboard to normalize the vertical (Y-axis) acceleration component by applying a +1 g offset, which is then packaged for wireless transmission. This prepares raw acceleration values for further analysis. In the current implementation, the calculation of higher-level vibration characteristics such as amplitude and variance would typically occur on the Central Monitoring Platform after receiving the offset-corrected acceleration data.

### 2.3. GPS Module

The GPS module provides time-stamped position data, including latitude and longitude, sampled at 1 Hz. It is connected to the ESP32 via a UART connection using the TX and RX pins [29]. Although the GPS data is slower than the IMU, it is still crucial for assigning the detected anomalies to specific geographical locations.

In this system, the GPS module has the task of recording geographical coordinates—in particular latitude and longitude—which are sampled at a predefined frequency. These coordinates are critical for correlating vibration measurements with specific locations on the track and allow for the accurate identification and mapping of potential structural anomalies. The included serial communication interface facilitates the acquisition of real-time position data. Although the GPS module typically operates at a lower sampling rate compared to the IMU, the position data it provides is sufficiently accurate to identify the locations of potential structural anomalies.

The time offset between the GPS (1 Hz) and IMU (60 Hz) readings was evaluated to be a maximum of one second, as each IMU sample is tagged with the last received GPS coordinate [7], which creates a maximum potential time offset of just under one second. This offset is considered negligible for the practical application of localizing track anomalies. For example, a tram moving at a typical urban speed of 40 km/h (approximately 11 m/s) would travel a maximum of 11 m within this time window. This meter-level accuracy is sufficient for maintenance crews to identify a flagged anomaly zone for subsequent inspection and does not impact the system’s utility. Furthermore, since our validation method relies on the consistency of an anomaly’s location across multiple passes, any systematic offset is accounted for, ensuring the system can reliably pinpoint the same location. The ability to precisely localize detected anomalies transforms raw vibration data into actionable information that is essential for targeted maintenance and emergency response. Without precise spatial mapping, even highly accurate vibration data would be of limited practical use for infrastructure management.

### 2.4. Data Transmission via MQTT

Sensor data from the IMU and the GPS are summarized in a structured JSON message and published to an MQTT broker via Wi-Fi. The ESP32 connects to a predefined topic so that a central server can subscribe and receive updates in real time. This architecture supports scalable integration with cloud-based dashboards, digital twins and alerting systems. The data can be processed remotely to detect and visualize anomalies and analyze historical trends.

For technical completeness, the system was configured to balance near real-time data flow with network efficiency. The ESP32 batches sensor readings and publishes them to the MQTT broker at a frequency of 10 Hz (every 100 ms). Each MQTT message is a JSON object containing the latest available GPS coordinate and a batch of six time-stamped accelerometer readings collected during that interval. The typical packet size of each JSON message is approximately 250–300 bytes.

This hardware-software architecture supports continuous, real-time condition monitoring while minimizing local storage requirements, improving scalability and enabling integration into the smart city infrastructure.

## 3. Vibration Data Processing and Statistical Analysis

### 3.1. Characterization of Vibrations and Sources of Track Anomalies

Vibrations are oscillating movements around an equilibrium position. In transportation infrastructure, undesirable vibrations are often caused by structural anomalies such as worn tram sections, loose fastenings, surface deterioration or disruptive soiling. These anomalies lead to transient mechanical excitations during vehicle transit, resulting in non-periodic vibrations.

**Figure 3:**
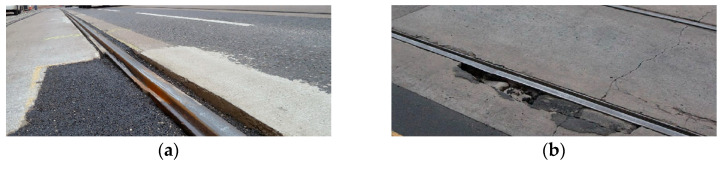
Illustrative examples of railway track defects: (**a**) degradation of road surface; (**b**) significant structural defect.

This study focuses specifically on externally induced, non-periodic vertical vibrations associated with infrastructure anomalies. This selective focus isolates signals indicative of structural anomalies, reduces background noise from routine vehicle operation, and improves the accuracy of subsequent anomaly detection. Recent research, such as studies on estimating track anomalies from car body vibrations using data-driven approaches [20], further emphasize the value of isolating these specific signals for effective track management. Figure 3 provides representative examples of the anomalies our system is designed to identify, such as surface degradation and significant structural defects before they become visually obvious.

### 3.2. Data Collection and Preprocessing Techniques

Vibration raw data is acquired by a triaxial MEMS accelerometer with a sampling rate of 60 Hz, chosen to capture the frequency range typically associated with vehicle-track interactions. Each sample comprises three acceleration components (X, Y and Z axis) and is accompanied by geographical coordinates (latitude and longitude) determined via GPS.

The Message Queuing Telemetry Transport protocol is used for real-time data transmission from distributed sensors. Its lightweight publish/subscribe messaging model is ideal for resource-constrained devices and unreliable networks, making it critical for continuous, scalable infrastructure monitoring and dealing with intermittent connectivity in urban field deployments. Sensor nodes act as MQTT clients and publish vibration data to a central MQTT broker. This broker then distributes the data to subscribed applications for real-time processing and storage. This architecture improves scalability, reduces network overhead and supports asynchronous communication, which is critical for dealing with intermittent connectivity in field deployments.

Data is stored in a structured text format and post-processed using Python 3.11 (Python Software Foundation) due to its flexibility and robust data processing libraries. Unlike many signal processing pipelines, a low-pass filter such as a moving average was deliberately omitted in our pre-processing [30]. The primary goal of this study is to detect the sharp, high-amplitude transient events characteristics of track defects. Any low-pass filter can slightly attenuate the peak amplitude of these signals, and we chose to analyze the unfiltered data to preserve the true peak magnitude of these critical events. Our anomaly detection method relies on a very stringent ±6σ threshold. This high threshold is, by its nature, extremely effective at rejecting low-level, random sensor noise, making a preliminary filtering stage redundant for this specific application. Pre-processing begins with the normalization of the vertical acceleration component. Since this axis is oriented in the opposite direction to the gravity vector, it is registered at approximately −1 g under static conditions. An offset of +1 g is applied to center the vibration signal around the zero point, which increases the clarity of the vertical displacement signals and facilitates subsequent statistical analysis.

Although the system is currently optimized for short-term, real-time anomaly detection, its design is inherently extensible to long-term deployments. Continuous data collection over weeks or months would enable trend-based diagnosis and predictive maintenance, supporting proactive infrastructure management. This capability aligns in line with the increasing focus on data-driven predictive maintenance in tram transportation [31,32], and represents a key objective for future development.

### 3.3. Statistical Analysis for Anomaly Detection

Anomaly detection assumes that vertical vibration data follows a distribution in the absence of anomalies. From this distribution, the empirical mean and variance are computed. In the analyzed dataset, the Y-axis mean was approximately 0.015 g with low variance, indicating consistent sensor response.

To identify outliers, a statistical threshold of ±6σ from the mean is applied. This criterion provides a confidence level greater than 99.999998, significantly reducing the likelihood of false positives, which are particularly costly in critical infrastructure monitoring scenarios. This high-threshold approach aligns with Six Sigma methodologies commonly employed in quality assurance and predictive diagnostics, offering robustness against noise and benign transients. However, this conservative approach may underreport emerging or subtle degradation. In this study, an anomaly is defined as a statistical outlier in the vibration data that exceeds the ±6σ threshold. Such anomalies are potential indicators of physical faults or systemic degradation in the track infrastructure. A direction for future work includes the development of adaptive thresholds. This approach, supported by recent reviews on machine learning for tram monitoring [33], will involve creating a dynamic model where the detection threshold adjusts in real-time based on operational variables such as vehicle speed, load, and track geometry. By incorporating context-aware statistical modeling and potentially leveraging advanced techniques like those in deep learning for anomaly detection, the system could improve early detection capabilities while maintaining a low false alarm rate.

Although vibration signals from tram–track interaction may deviate from perfect normality and strict stationarity, the choice of a very conservative threshold ensures that only the most extreme deviations are flagged as anomalies. In practice, this makes the approach robust against heavy-tailed distributions, short-term non-stationarities, and benign operational transients that could otherwise produce nuisance alarms. This rationale aligns with Six Sigma methodology in quality control, where ±6σ limits are employed specifically to minimize the probability of false alarms or defects per million opportunities in high-cost processes [34,35].

The operational motivation for this choice lies in the cost of false alarms in critical infrastructure monitoring. Each false alert requires manual inspection and may disrupt operations; therefore, maintaining an extremely low false positive rate is essential for system acceptance. Similar reasoning has been highlighted in railway condition-monitoring reviews, which caution that excessive false alarms lead to alarm fatigue and reduced system trustworthiness [36,37]. By adopting a stringent threshold, our method prioritizes operational reliability and robustness, providing a transparent baseline framework for anomaly detection. An anomaly is thus defined in this study as a statistical outlier in the vibration data exceeding the ±6σ threshold, which is interpreted as a potential indicator of physical faults or systemic degradation in the track infrastructure. While this conservative choice may underreport subtle early-stage degradations, it represents a deliberate trade-off favoring reliability over sensitivity at the initial deployment stage. Future work will explore adaptive thresholds that integrate operational variables such as vehicle speed, load, and track geometry, as recommended in recent surveys on data-driven railway monitoring [37,38].

**Figure 4:**
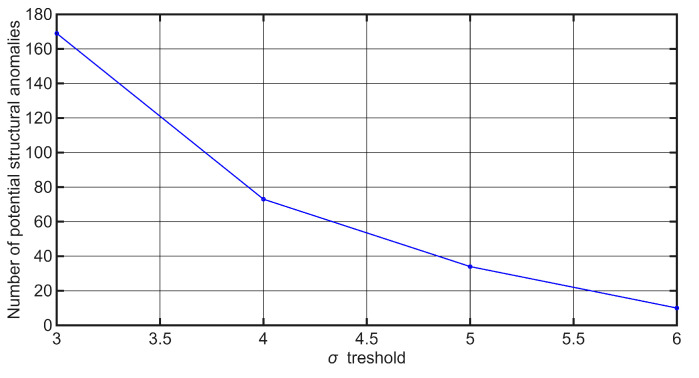
Number of outliers/number of potential structural anomalies as a function of the ±sigma threshold.

A detailed statistical analysis was additionally performed on a dataset comprising 1500 recorded samples. The results revealed that the number of outliers strongly depends on the chosen sigma threshold: 169 samples fall outside ±3σ, 73 outside ±4σ, 34 outside ±5σ, and 10 outside ±6σ. Considering practical experience, it is highly improbable that as many as 169 values represent true anomalies, which clearly demonstrates that the ±3σ criterion would lead to an excessive number of false positives. For this reason, the ±3σ threshold is not favorable for our application, and stricter thresholds (±4σ and beyond) provide a more balanced trade-off between sensitivity and reliability. This trend is further illustrated in Figure 4, where the dependence of the number of outliers on the sigma threshold is presented.

## 4. Real-World Measurement Results and System Validation

The following case studies were analyzed using a systematic, three-step process to identify, locate, and validate potential track anomalies from the collected vibration data. First, detection was performed by applying a stringent ±6σ statistical threshold to the vertical acceleration data; any data point exceeding this threshold was objectively defined as a potential anomaly. Second, localization was achieved by matching each detected anomaly with the most recent GPS coordinate for precise spatial mapping. Finally, the reliability of a potential anomaly was confirmed through validation, which required the anomaly to be repeatable—consistently detected at the same geographic coordinate across multiple vehicles runs. This framework was applied to the data from the following sections.

### 4.1. Experimental Setup and Data Acquisition Environment

To validate the proposed VTAD system, a prototype for detecting anomalies on the tram infrastructure was developed and subjected to rigorous testing under real operating conditions. The field tests were conducted on a CROTRAM TMK 2200 tram, which has a mass of approximately 40.4 tons and a maximum operational speed of 70 km/h.

**Figure 5:**
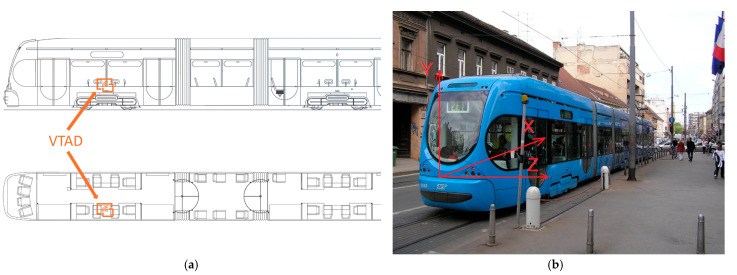
VTAD position in the tram: (**a**) top and side view; (**b**) Sensor axis orientation: the tram moves in the direction of the X−axis.

**Figure 6:**
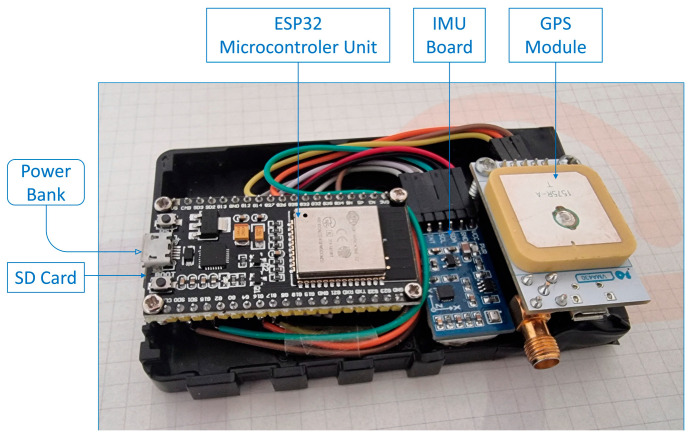
VTAD device prototype.

To validate the proposed VTAD system, the VTAD prototype was subjected to rigorous testing under real operating conditions. For this feasibility study, the sensor was placed on the cabin floor above the axle at the rear of an operational tram to facilitate rapid, non-invasive deployment on operational vehicles. Future work could explore mounting sensors directly on the bogie to capture vibration signals with less damping, though this would present greater installation and logistical challenges. As illustrated in the schematic in Figure 5a, the unit was positioned in the cabin on the floor directly over the rear axle to ensure maximum sensitivity to track-induced vibrations. The unit, housed in a protective casing as shown in the photograph in Figure 6, was securely affixed to the floor, and power was supplied by a portable USB power bank. The orientation of the sensor’s measurement axes is detailed in Figure 5b. The subsequent time-series graphs display the recorded acceleration in g on the y-axis versus the sample number on the x-axis.

### 4.2. Case Study 1: Tram Route at Section of Šubićeva Street, Zagreb

**Figure 7:**
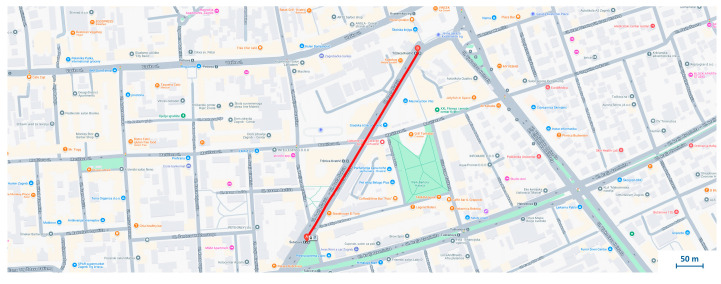
Section of Case Study 1 used for vibration analysis.

The analysis was carried out on a section of Šubićeva Street in Zagreb, as shown in Figure 7. The measurement data from this section was used to create the corresponding diagrams.

#### Axis-Specific Acceleration Analysis

Acceleration data collected along the Case Study 1 section was analyzed along three orthogonal axes to assess typical tram dynamics and identify potential infrastructure anomalies. The sensor axis orientation used for data collection is illustrated in Figure 5, with the X, Y, and Z axes corresponding to longitudinal, vertical, and lateral movements, respectively.

**Figure 8:**
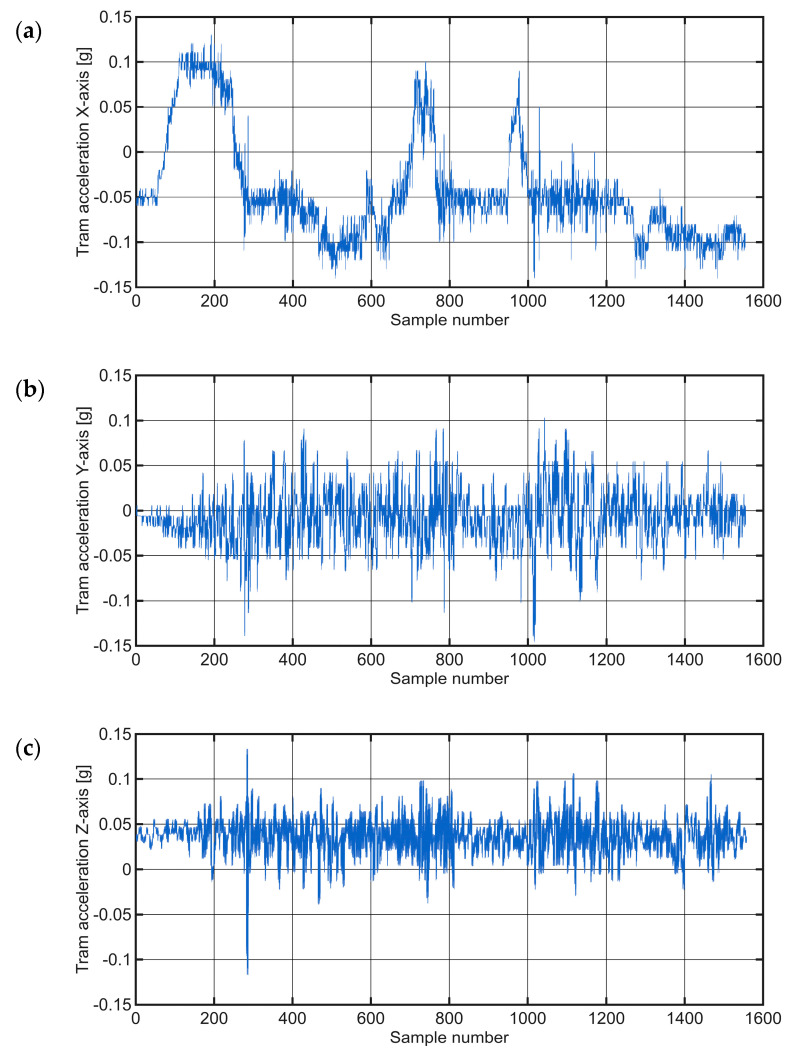
Raw time-series data of tram acceleration in the: (**a**) X−axis; (**b**) Y−axis; (**c**) Z−axis.

X-axis acceleration: The X-axis is aligned with the tram direction of travel and reflects longitudinal acceleration and deceleration. Figure 8a illustrates a minimum signal activity in the interval from 0 to 80 corresponds to the standstill of the tram at traffic signals or stops. Acceleration phases appear as upward slopes (e.g., sampled values 80–280), while deceleration is reflected in downward trends. When driving steadily, the values oscillate around the zero point. Minor baseline shifts (e.g., oscillation at −0.05 g) are due to slight misalignment of the sensor and are considered negligible.

Y-axis acceleration: The Y-axis, which is oriented vertically and in the opposite direction to the gravitational vector, shows −1 g when stationary. To facilitate interpretation, an offset of +1 g was applied, effectively centering the signal around the zero point, as shown in Figure 8b. In normal operation, the values are typically between −0.1 g and 0.1 g. Peak values exceeding the ±6σ threshold may indicate anomalies such as track anomalies or structural problems. Repeated measurements over the same section improve the system’s ability to distinguish between real anomalies and random disturbances.

Z-axis acceleration: The Z-axis corresponds to the lateral acceleration aligned with the tram doors. As depicted in Figure 8c, this axis detects lateral vibrations that can be caused by track curves, gradients or uneven wear. Due to the limitations of rigid sensor mounting along this axis, the results must be interpreted with caution. Nevertheless, the signal generally remained within the range of −0.2 g to 0.15 g, indicating normal lateral dynamics, as no significant deviations were detected during this ride.

Table 1 provides a quantitative overview of the system’s basic behavior during normal tram operation by summarizing the minimum, average, and maximum acceleration values recorded for each axis.

This study segment illustrates the expected tram motion dynamics and indicates that the Y-axis is the most sensitive to structural anomalies, however in this case no critical anomalies were observed. The Y-axis is considered the most sensitive because vertical accelerations provide the most direct measurement of the impacts caused by track discontinuities such as worn joints or surface defects, whereas longitudinal (X-axis) and lateral (Z-axis) accelerations are more strongly influenced by operational dynamics like braking and turning.

### 4.3. Case Study 2: Tram Route from Dubrava to the Kvaternik Square, Zagreb

**Figure 9:**
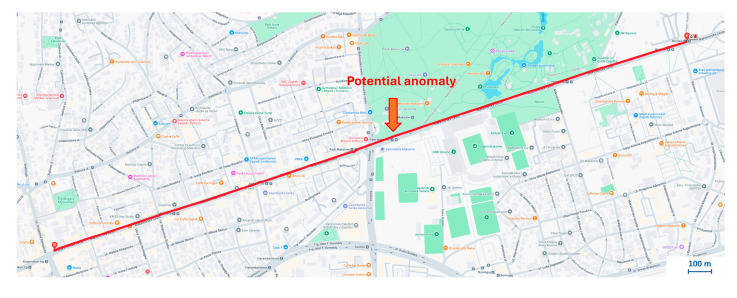
Section of the tram route from Dubrava to the Kvaternik Square, with the indicated location of potential anomaly.

To further validate the system, data was collected along a 2.69 km tram route from Dubrava to Kvaternik Square, generating more than 15,000 samples in about 10 min, Figure 9. Although subjectively smooth, a detailed signal analysis was performed.

**Figure 10:**
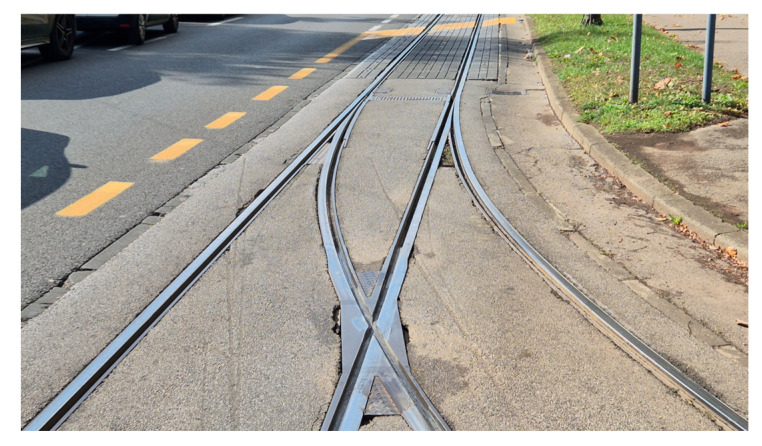
On-site photograph of the track defect corresponding to the consistently detected anomaly in Case Study 2.

The Y-axis acceleration data were modeled with a normal distribution, with a calculated mean of 0.0146 and a variance of 0.0473. Based on the ±6σ threshold, amplitudes exceeding ±0.288 g were classified as statistical outliers, possibly indicating track anomalies. Several such outliers were observed in the first measurement run. It is noteworthy that one anomaly was consistently detected at the specific geographic coordinate (45.81927, 16.01457) during measurement runs. This location corresponds to a tram junction, Figure 10. This condition causes high-amplitude impacts as the tram’s wheels traverse them, appearing as clear disturbances in the vertical vibration signal. The consistent detection of this event confirms the system’s ability to reliably identify and locate significant structural features that are known sources of mechanical stress and vibration.

**Figure 11:**
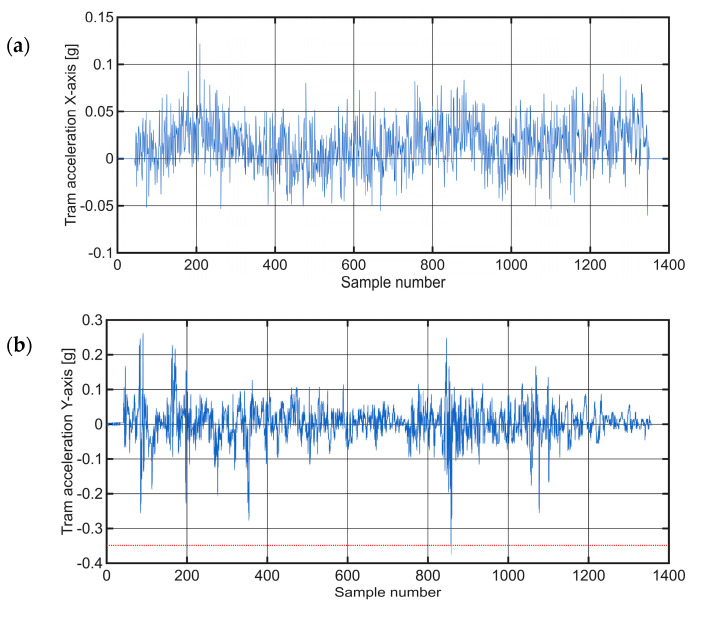

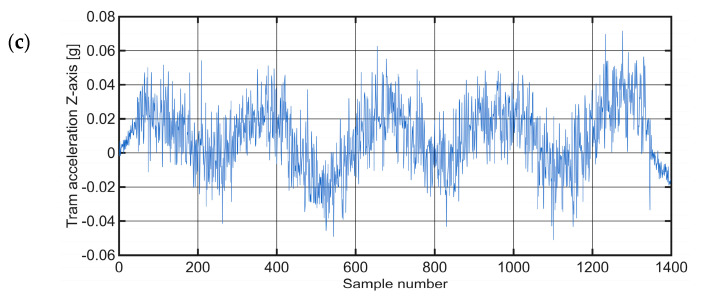
Raw time-series data of tram acceleration in the: (**a**) X−axis; (**b**) Y−axis; (**c**) Z−axis for Case Study 2 route. The red dotted line in (**b**) represents the ±6σ threshold.

**Figure 12:**
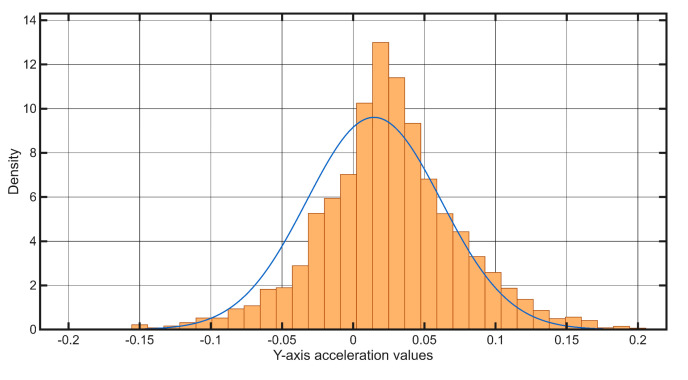
Histogram of Y−axis acceleration values measured during the test for the Case Study 2 route, overlaid with a fitted Normal distribution density function.

Figure 11 shows the raw time-series data of tram acceleration in all three axes, while Figure 12 presents the distribution of values on the Y-axis and the corresponding normal distribution fit.

To support the spatial validation, the GPS coordinates of the sample were recorded and processed using MATLAB (R2023b, Version 9.14, The MathWorks Inc., Natick, MA, USA). Despite minor deviations due to signal noise and GPS errors, the position of the repeated anomaly remained consistent within a tolerable margin of a few meters, confirming the reliability of the system’s localization capability.

**Figure 13:**
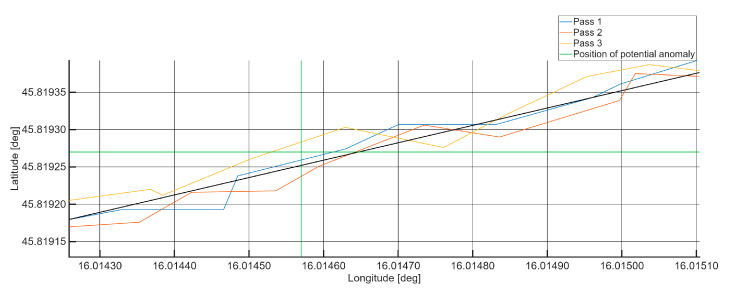
Spatial trajectories of the Global Positioning System coordinates recorded by the VTAD prototype system across three consecutive measurement passes.

Figure 13 illustrates the strong spatial convergence of the GPS trajectories recorded during three independent measurements passes by the VTAD prototype, specifically highlighting the area around a detected anomaly. This close alignment of the three traces clearly demonstrates the high level of positional repeatability achieved and validates the operational stability of the VTAD sensor configuration during field testing.

This established spatial consistency is critical for ensuring that detected vibration signatures can be reliably compared and aggregated across repeated measurements. Furthermore, it shows that local variations in the recorded coordinates remain well contained within the nominal accuracy envelope of the prototype receiver. These observations confirm that the residual positional uncertainty is negligible and does not compromise the accurate localization or interpretation of vibration anomalies. Overall, the prototype system achieves the required spatial precision for consistent detection and analysis of track irregularities, meeting established positional accuracy standards for railway monitoring applications [39,40].

This case clearly demonstrated the system’s ability to reliably identify and locate a persistent track anomaly through repeated measurements and confirmed the practical application of the system in distinguishing real structural problems from transient noise, unlike the first case which showed more typical operational patterns.

### 4.4. Case Study 3: Tram Turn at Kvaternik Square, Zagreb

**Figure 14:**
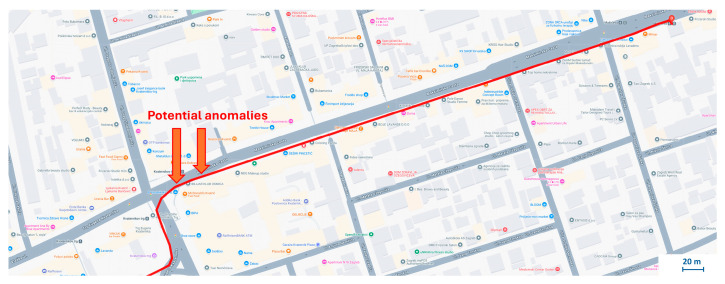
Section of curved tram turn in Case Study 3, with the indicated locations of potential anomalies.

Another critical section that was analyzed was the tram curve at Kvaternik Square in Zagreb, as shown in Figure 14. Tram junctions and crossings over curved tracks naturally generate stronger vibrations than straight sections. This operational context requires an adaptive threshold for anomaly detection that can account for the expected variations in vibration patterns depending on the operating environment. This requirement emphasizes the importance of contextual awareness for accurate anomaly detection in different operational scenarios. A static threshold applied universally would lead to frequent false alarms in areas with naturally higher vibration profiles. Such an adaptive threshold could be implemented by dynamically adjusting the sigma multiplier based on factors such as vehicle speed, track geometry or historical data for specific sections, allowing the system to be optimized for different maintenance objectives.

**Figure 15:**
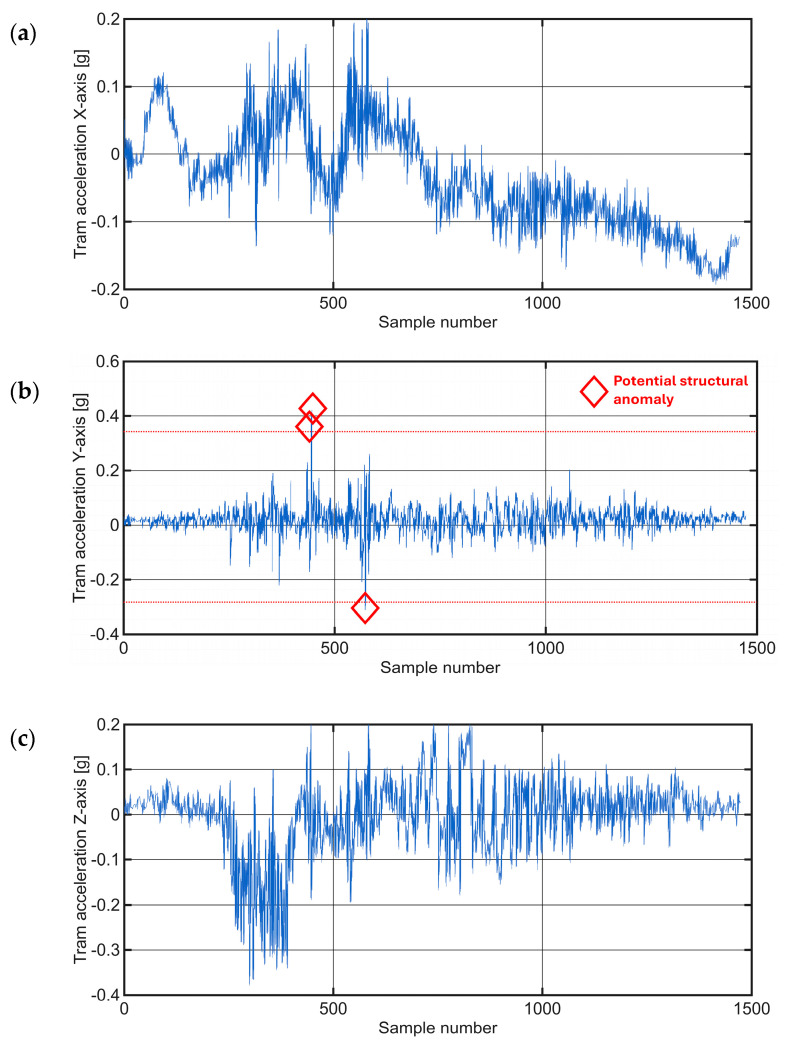
Raw time-series acceleration data for a tram traversing a curved section in the Case Study 3, presented along the: (**a**) X−axis; (**b**) Y−axis; (**c**) Z−axis. The red dotted line in (**b**) represents the ±6σ threshold.

The section of track examined in this Case Study spanned approximately 430 m, yielding around 1500 data samples. A comprehensive investigation of all three acceleration axes provided unique insights into the dynamics of the tram’s movement during the curve, as shown in Figure 15. In the initial phase of the curve, a gradual acceleration of the X-axis values was observed. At the same time, the Z-axis showed a negative acceleration, which corresponds to a rightward turning movement and the associated centripetal acceleration towards the center of the curve. Discontinuities in the track structure during the traverse appeared as clear disturbances in the Y-axis signal. In the last 1000 samples, the tram slowed down in preparation for stopping at a nearby stop. The software determined the mean (μ) for this section to be 0.0198 and the variance (σ^2^) to be approximately 0.049.

**Figure 16:**
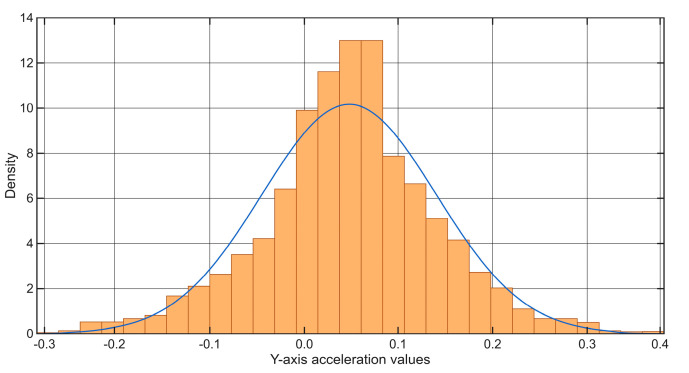
Histogram of Y−axis acceleration values measured during the tram’s traverse of the curved section overlaid with a fitted Normal distribution density function.

Figure 15b shows the amplitude values oscillating around zero. This visualization highlights three anomalies, marked with red diamonds which represent significant deviations from the normal vibration profile observed during the tram turn. The figure emphasizes discontinuities within the track structure that manifested as noticeable disturbances in the Y-axis signal. The histogram and the representation of the normal distribution for this section can be seen in Figure 16.

**Figure 17:**
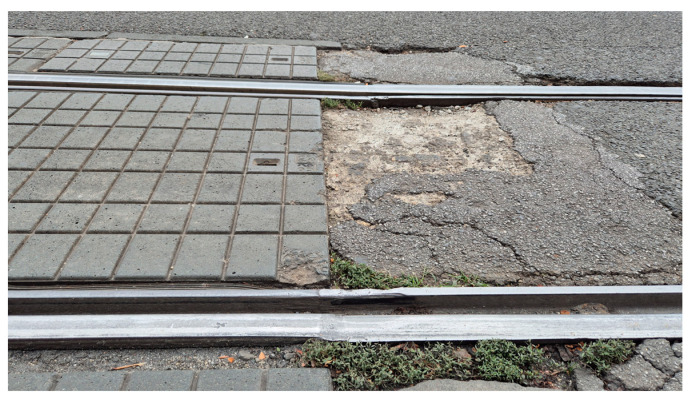
On-site photograph of the track defect corresponding to the consistently detected anomaly in Case Study 3.

The system flagged three significant deviations from the expected vibration profile, as marked in Figure 15b. A follow-up on-site inspection confirmed that these high-amplitude events correspond to physical discontinuities in the track structure, specifically areas of significant pavement degradation around track joints, as shown in the photograph in Figure 17. This finding demonstrates the system’s ability to correctly identify genuine infrastructure issues even within a complex, high-vibration environment like a track turn.

This case study highlighted the inherent challenge of distinguishing genuine anomalies from increased but expected vibrations that occur in certain track geometries, such as curves. This observation highlights the critical need for adaptive thresholds in anomaly detection, as static thresholds applied universally would lead to frequent false positives in areas with naturally higher vibration profiles. This is in stark contrast to Case Study 2, where a clear and consistent structural anomaly was reliably detected under more uniform track conditions.

### 4.5. Case Study 4: Vukovarska Street, Zagreb

**Figure 18:**
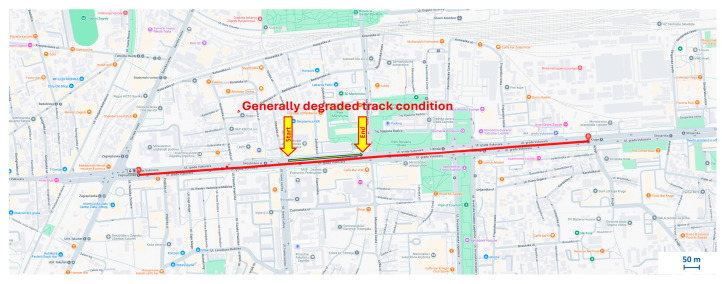
Section of Case Study 4 with marked segment of interest.

Among the various routes examined in this study, one section of Vukovarska Street in Zagreb exhibited a distinct pattern of successive high amplitude acceleration values. This section, highlighted in Figure 18, raised the suspicion that this was not isolated anomaly, but rather a general deterioration in track condition, possibly indicative of an outdated or heavily worn tram infrastructure.

This ability to distinguish between isolated faults and systemic degradation is critical for strategic maintenance planning, as it indicates whether a quick, targeted repair is sufficient or whether a more comprehensive infrastructure overhaul is required.

**Figure 19:**
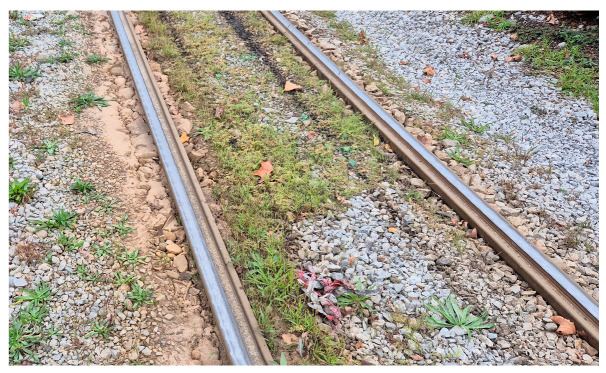
On-site photograph illustrating the general track conditions in Case Study 4.

Unlike the isolated, sharp anomaly in Case Study 2, the system detected a pattern of consistently elevated vibration values along a longer segment of Vukovarska Street. This indicates systemic degradation rather than an isolated fault. An on-site visual inspection confirmed this assessment, revealing conditions such as uneven ballast, vegetation growth, and overall wear along this section, as illustrated in Figure 19. These factors collectively contribute to the sustained high vibration levels, highlighting the system’s ability to identify areas requiring general maintenance attention for predictive purposes.

**Figure 20:**
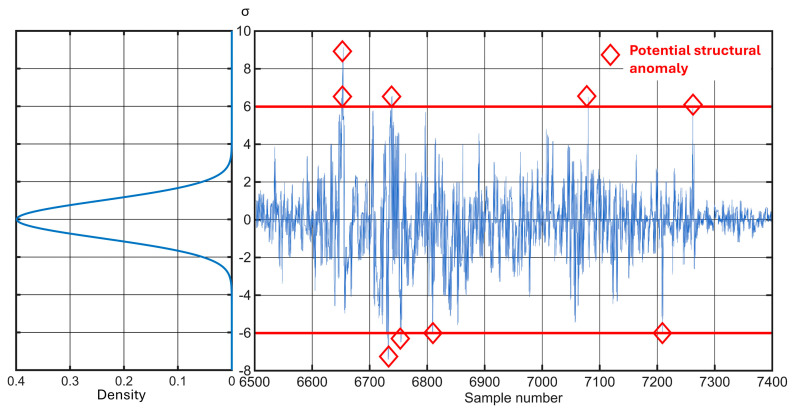
Anomaly detection on a degraded track section (Case Study 4) using the ±6σ threshold represented by the red line.

The amplitude peaks associated with the above-mentioned anomalies are shown in the graph in Figure 20. This visualization overlays the raw sensor data with a normal distribution curve to illustrate how the thresholds for anomaly detection are determined. The distribution is centered around the empirical mean; deviation intervals are defined as multiples of the standard deviation (σ). All amplitude values that fall within the interval [−6σ, +6σ] are considered statistically normal and negligible. Conversely, values that fall outside this strict range, meaning they deviate from the mean by more than six standard deviations—can be classified as potential structural anomalies with 99.999998 confidence. This strict threshold ensures that only highly significant and probable track deterioration is flagged, minimizing false alarms and focusing maintenance efforts on critical areas.

**Figure 21:**
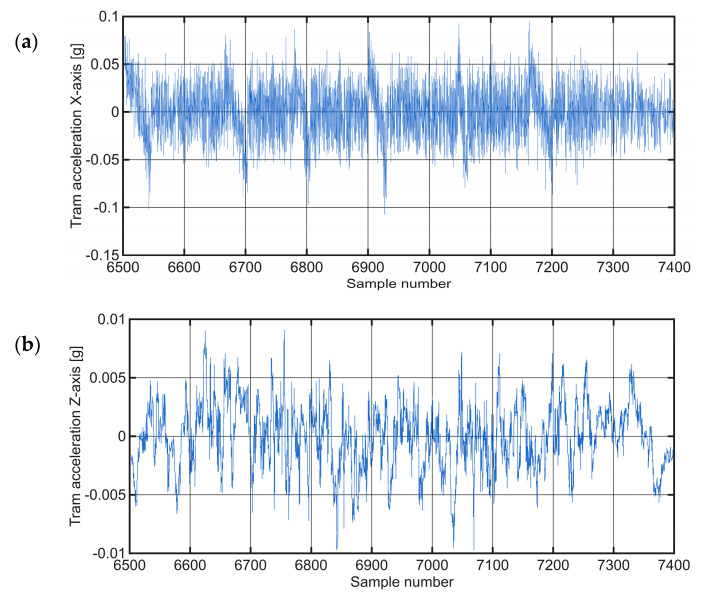
Raw time-series acceleration data on a degraded track selection (Case Study 4), presented along the: (**a**) X−axis; (**b**) Y−axis.

The corresponding accelerations for the same section of the route (Case Study 4), for which the observed anomalies were described, are shown in Figure 21, illustrating the acceleration components along the X−axes and Y−axes.

Unlike the previous cases, which focused on individual anomalies or baseline behavior, this case study illustrates the system’s potential to detect widespread track degradation. This distinction is crucial as it enables the development of differentiated maintenance strategies that allow the strategic deployment of either targeted repairs for isolated faults or comprehensive infrastructure overhauls for systemic deterioration.

## 5. Discussion: Advantages, Broader Applications, and Future Directions

The proposed vibration-based anomaly detection system offers several key advantages over traditional infrastructure inspection techniques. First and foremost are its cost efficiency and compactness. Although conventional diagnostic vehicles offer high measurement accuracy, they are expensive to purchase and operate. In contrast, the proposed system uses off-the-shelf, low-cost components such as MEMS accelerometers, GPS modules and microcontrollers, enabling scalable deployment at a fraction of the cost [41].

Secondly, the system supports real-time and continuous monitoring. Conventional methods rely on regular manual inspections or scheduled diagnostic runs, which introduce latency in anomaly detection. In contrast, the proposed system works passively during normal vehicle operation and enables uninterrupted data collection without additional vehicle downtime. This supports the shift from periodic maintenance to predictive maintenance, in line with current trends in smart infrastructure management [7,42].

Third, the system is modular and scalable and can be integrated into different types of vehicles—trams, buses or service vehicles—transforming an urban fleet into a distributed sensor network. This crowdsensing approach eliminates the need for dedicated diagnostic units and instead utilizes the inherent mobility of existing fleets, increasing both coverage and efficiency [1,7].

Finally, the system improves the reliability of diagnostics through iterative validation of anomalies. Locations where abnormal vibration signatures persist across multiple crossings can be flagged with high confidence, significantly reducing the number of false alarms. This capability supports targeted, data-driven maintenance interventions and increases infrastructure safety [7].

### 5.1. Integration with Intelligent Transport Systems and Smart City Infrastructure

In addition to infrastructure diagnostics, the system offers considerable potential for integration into intelligent transportation systems and smart city concepts. Real-time data streaming via modern 5G networks enables low-latency communication with centralized platforms, facilitating dynamic updates of the digital twins of urban infrastructure. These digital representations support predictive maintenance, optimized asset management and coordinated emergency response.

By embedding the system into public transportation, cities can establish a passive, high-resolution sensor layer that continuously captures structural health metrics. Integration with cloud services, automated alert systems and AI-driven anomaly detection models further enhances system capabilities [9,10].

Integrating this data into broader smart city platforms—such as geographic information system (GIS) layers, traffic management systems or maintenance planning software—supports cross-domain optimization. For example, predictive models based on risk profiles or real-time congestion forecasts can prioritize interventions.

However, widespread application requires careful attention to data management and cyber security. The continuous collection of vehicle trajectories and vibration data raises concerns about data ownership, user privacy and the possibility of unauthorized access. Although not directly the subject of this study, future research must address data privacy by design, encryption protocols and secure cloud architectures to ensure system trustworthiness and regulatory compliance.

### 5.2. Proposed Hardware and Software Enhancements

Planned enhancements aim to improve detection accuracy, analytical capabilities and system integration. At the hardware level, future versions could include high-precision accelerometers and gyroscopes to correct orientation shifts and improve the accuracy of acceleration measurements. The use of multiple sensors per vehicle on the front and rear axles could provide spatial redundancy and improve the accuracy of anomalies localization.

On the software and analytics side, the integration of cloud-based platforms for real-time processing can enable automatic updates of GIS dashboards and visualizations of the digital twin with color-coded overlays to show anomaly severity. This functionality facilitates intuitive interpretation by engineers and maintenance personnel.

Advanced machine learning techniques, including transformer-based models and deep neural networks [3,33], offer promising opportunities for adaptive anomaly detection. These models could outperform threshold-based methods by learning context-dependent vibration patterns under different operating conditions. In addition, research in multimodal data fusion—combining vibration signals with computer vision and environmental sensing—could enable holistic diagnosis of infrastructure health [6].

To further contextualize system performance, comparative assessments with traditional and modern inspection methods are recommended. While dedicated high-end systems may offer finer spatial resolution or frequency domain analysis, the proposed solution is characterized by its cost-effectiveness, real-time coverage and application scalability. Future work should quantify detection accuracy, false positive rates and operational impact in terms of maintenance effectiveness.

In addition to presenting the current anomaly detection framework, this study highlights several directions for future work. First, the adoption of adaptive thresholds will be explored, where detection limits are dynamically adjusted according to operational conditions such as tram speed, track curvature, and vehicle load. This context-aware approach can reduce the likelihood of irritant alarms in track sections that naturally generate higher variability. Second, we will investigate the inclusion of frequency-domain features derived from vibration signals. While the present analysis focuses on time-domain thresholds, spectral features such as dominant frequency bands, harmonic content, and energy distribution have shown strong diagnostic potential in railway vibration studies. Their integration may improve sensitivity to emerging or subtle degradation patterns.

As future work, we plan to investigate adaptive thresholding strategies based on the current dataset. Unlike the fixed statistical thresholds applied in the present study, adaptive approaches would allow the system to dynamically adjust sensitivity in response to operational conditions such as vehicle speed, track curvature, and load variations. By exploiting contextual information embedded in the vibration data, the system could continuously optimize its decision boundaries, thereby minimizing the likelihood of false positives while ensuring timely identification of structural anomalies. Such a data-driven adjustment mechanism would not only improve reliability but also enhance the scalability of the proposed monitoring approach for broader deployment in urban rail networks.

Moreover, exploring adaptive thresholds could provide the foundation for more advanced techniques, including the integration of machine learning and deep learning models that can automatically learn relationships between operational parameters and anomaly signatures. This would open the possibility of developing hybrid detection frameworks that combine statistical rules with model-based predictions, ultimately leading to more accurate and robust monitoring solutions. In addition to reducing human intervention in parameter tuning, such developments could strengthen the applicability of the system in future smart city ecosystems, where railway infrastructure monitoring will increasingly be connected with IoT platforms and digital twin environments.

## 6. Conclusions

This study demonstrates the feasibility and effectiveness of a microcontroller-based vibration sensor system for the detection of structural anomalies in tram and road infrastructure. The developed prototype of Vibration-based Track Anomaly Detection (VTAD) system utilizes low-cost, off-the-shelf components. VTAD successfully captured relevant vibration signatures and identified potential anomaly locations using a ±6σ threshold for anomalies and repeated measurements along Zagreb tram routes.

The system offers several key advantages over traditional inspection methods: it is compact, cost-effective and scalable, and enables deployment on public transportation without the need for dedicated diagnostic vehicles. This passive sensor approach enables continuous monitoring of infrastructure in real time and represents a shift towards predictive maintenance strategies that can improve transportation reliability, reduce operating costs and increase passenger safety.

Future enhancements to the VTAD system include the integration of advanced technologies such as 5G connectivity, cloud-based analytics, urban digital twins, and AI-driven anomaly detection. These advancements hold significant potential for expanding VTAD’s capabilities. By enabling real-time diagnostics and proactive infrastructure management, this proposed approach contributes significantly to the development of intelligent transportation systems and supports the broader vision of resilient, data-driven urban mobility.

## Figures and Tables

**Table 1:** Acceleration statistics by axis.

Axis	Min (g)	Avg (g)	Max (g)
X	−0.17	0.05	0.27
Y	−0.77	0.0135	0.50
Z	−0.36	0.00	0.29

## Data Availability

The original contributions presented in this study are included in the article.
